# News and Coming Events

**Published:** 1908-03-14

**Authors:** 


### NEWS AND COMING EVENTS.

His Royal Highness the Prince of Wales, at the Council of the Duchy of Cornwall, held on March 5, was pleased to appoint to the office of Sheriff for the County of Cornwall John Cosmo Stuart Rashleigh, M.D., of Menabilly, Cornwall.

Mr. A. Leslie Wright, of Butterley Hall, Derbyshire, has offered to build and equip wards for children in the Derbyshire Royal Infirmary in memory of his late wife, at a cost of between £7,000 and £8,000.

In reply to a question asked in the House of Commons by Sir F. Powell on March 5, Mr. Haldane said: Medical officers of the Volunteers will have the option of transferring to the Royal Territorial Medical Corps or of remaining regimental officers. Those who transfer will be permitted to retain the uniform of their former unit until worn out. The compound title will be abolished in the case of those who join the Territorial Medical Corps. The appointment of Brigade Surgeon in the Territorial Force will lapse, as the Territorial Force will be organised on the same basis as the Regular Army, in which such appointment does not exist.

When the Royal Commission on Experiments on Animals was appointed, a meeting was held of delegates of scientific and medical societies, and a committee was formed (Professor Starling's committee) to ensure that the evidence as to the value and necessity of these experiments should be presented to the Royal Commission in proper order. The work [illegible] Professor Starling's committee came to an end when the Commission ceased to require further evidence. At a meeting of the committee it was decided to form a society, under the title of the Research Defence Society, to make generally known the fact about experiments on animals in this country, and to offer steady opposition to the anti-vivisection societies. Among those who have already joined the society are eminent men of science, leading hospital authorities, and distinguished physicians and surgeons. The Society hopes to be able to give information to all inquirers, to publish articles, to send speakers when required to debates, and to be of assistance to all who want to have a clear knowledge of the facts of the case. The Hon. Secretary is Mr. Stephen Paget, 70 Harley Street, W.

The annual meeting of the Court of Governors of King's College Hospital was held on February 27. The Rev. A. C. Headlam, D.D., the Principal of King's College, presided. The annual report stated that, although expenditure was closely w[illegible]hed and some gratifying economies had been effected, every legacy received during the year was absorbed by current expenses, and the year terminated with a deficit of £1,102. The report pointed out the magnitude and difficulty of the task of providing for annual maintenance, in addition to the large sum necessary for rebuilding. The Court approved the King's College (Transfer) Bill, which is being promoted by the Council of King's College and the Senate of the University of London, with the consent of the committee of management of the hospital. The Chairman referred to the removal of the hospital to South London, and said that tenders had just been signed for a part of the work, and that building would begin in a very short time. They had about £103,000 in hand towards the building scheme. This sum would last for some eighteen months, and he earnestly hoped that the further £100,000, the minimum amount necessary for the work, would be raised during that time.

The annual meeting of the Governors of the Chelsea Hospital for Women will be held on Thursday, March 19, the President (Lord Glenesk) in the chair.

The annual Court of Governors of the Mount Vernon Hospital for Consumption is appointed to be held at the offices, 7 Fitzroy Square, W., on Wednesday, March 18, at 4 p.m., the Marquis of Zetland, K.T., President, in the chair.

Sir Oliver Lodge presided at Birmingham on March 5 at a meeting to consider the question of providing assistance for Dr. Hall-Edwards, the $x$-ray specialist, who has had one hand amputated in consequence of injury caused by the Röntgen rays, and is in danger of losing the other. It was decided to raise a fund for the benefit of Dr. Hall-Edwards, the money to be invested, and a sum of £309 was subscribed in the room.

The death is announced from New York of Dr. Daniel Roosa, President of the New York Post-Graduate Medical School and a well-known ophthalmic surgeon. Dr. Roosa was born in 1838. He graduated in medicine at the University Medical College, New York, in 1860. He was assistant surgeon to the New York Volunteers during the Civil War. He had been professor of the University, City of New York, and the University of Vermont. He published several works on ophthalmic medicine.

Dr. Mary Scharlieb, surgeon to the women's department of the Royal Free Hospital, was presented with her portrait in recognition of her public and private work in the cause of charity and social progress. The portrait—painted by Mr. Hugh Rivière—has been subscribed for by Mrs. Scharlieb's personal friends to the number of nearly four hundred. The presentation took place at the house of Lady Frederick Cavendish on March 10. Mr. James Berry, who presided, unveiled the portrait and asked Mrs. Scharlieb's acceptance also of a book containing the signatures of the subscribers.

The present International Sleeping Sickness Commission is discussing measures for combating the disease. Considerable importance is attached to the fact that on this occasion (unlike the last) the leading delegate of each country represented will have full powers to sign the Act on behalf of his Government. The countries represented are Great Britain, France, Germany, Italy, Spain, Portugal, and the Congo Free State. Great Britain is represented by Lord Fitzmaurice, Sir Walter Foster, Sir Patrick Manson, Sir Rupert Bryce, Dr. Rose Bradford, Mr. Waldron Clarke, and Mr. Read. Germany is represented by Professor Koch, Herr von Jacobs, and Dr. Steudel; Italy by Professor R. Santoliquido and Professor Adolfo Gotta.

Hospitals in the County of London, or within nine miles of Charing Cross, desiring to participate in the grants made by King Edward's Hospital Fund for the year 1908 must make application before the 25th inst. to the Hon. Secretaries, 7 Walbrook, E.C. Applications will also be considered from convalescent homes and sanatoria for consumption which are situated within the above boundaries, or which, being situated outside, take a large proportion of patients from London. The scope of the operations of the Fund has been extended by the enlargement of the radius from seven to nine miles from Charing Cross, as well as by the admission to consideration of the claims of consumption satatoria situated in the country in which a large number of London patients are treated.

Dr. Edward Dillon Mapother died at the age of 72 on March 3 in London. He graduated as M.D. at the Royal University of Ireland in 1857, and took the Fellowship of the Royal College of Surgeons of Ireland in 1862. The greater part of his professional life was spent in Ireland. He was surgeon to St. Vincent's Hospital, and to St. Joseph's Hospital for Children in Dublin. He was professor of and examiner in physiology to the college, of which he was also President, and was Examiner in Surgery to the Queen's University of Ireland, and Surgeon in Ordinary to the Lord Lieutenant. He was the author of a text-book on physiology which passed through three editions, and of numerous works and papers dealing with dermatology and other medical subjects.

As we briefly announced last week, Sir Alfred Cooper, F.R.C.S., died at Mentone on March 3, aged 70 years, after a prolonged illness. Sir Alfred was educated at St. Bartholomew's Hospital, and became a member of the Royal College of Surgeons of England in 1861 and Fellow in 1870, having previously obtained the Fellowship of the Edinburgh College. He was for many years surgeon to the Lock Hospital, and he was also at different times surgeon to St. Mark's Hospital and to the West London Hospital. He was appointed Surgeon-in-Ordinary to the late Duke of Saxe-Coburg and Gotha (Duke of Edinburgh), and received various marks of distinction from Russian and German medical bodies. Sir Alfred was the author of important works upon the surgery of venereal disease. He had been Vice-President of the Royal College of Surgeons, and was a member of many medical and scientific societies. Sir Alfred married, in 1882, Lady Agnes Duff, daughter of the fifth Earl of Fife, and he was knighted in 1902.

The executive committee of the Society for the Destruction of Vermin, which was constituted at a public meeting on January 10, has decided upon the following policy :— The object of the Society is the destruction of vermin, but its chief action will be directed in the first instance against rats. A scientific committee has been formed, including Sir Patrick Manson, Dr. Sandwith, Mr. Frank Beddard, Mr. R. T. Pocock, Mr. E. E. Austen, Mr. James Cantlie, Sir Lauder Brunton, Prof. Wm. R. Smith, Dr. Carl Prausnitz, Dr. L. Sambon, and Dr. Hammond Smith, to conduct research and experimental work, while the actual destruction will be organised by an active committee. The Society does not commit itself to any one method of destruction or any particular specific, but holds itself open to give all methods and specifics a fair trial and to apply them according to circumstances. It is proposed to establish branches and provincial centres of the Society and to co-operate with existing organisation. It has been decided to apply for a certificate of incorporation under Board of Trade license as a non-profit-making society. The Society has no interest whatever in any specific for the destruction of rats or other vermin. The council of the Royal Institute of Public Health have allowed the Society to use their premises as a temporary address. The Society will endeavour to secure Government, municipal, and other public aid.

